# Acupuncture Treatment for Nocturnal Crying in Pediatric Patients: A Systematic Review of Clinical Studies

**DOI:** 10.3389/fped.2021.647098

**Published:** 2021-07-14

**Authors:** Lin Ang, Eunhye Song, Hye Won Lee, Jung Tae Kim, Eunseop Kim, Myeong Soo Lee

**Affiliations:** ^1^Clinical Medicine Division, Korea Institute of Oriental Medicine, Daejeon, South Korea; ^2^Korean Convergence Medicine, University of Science and Technology, Daejeon, South Korea; ^3^Global Strategy Division, Korea Institute of Oriental Medicine, Daejeon, South Korea; ^4^Herbal Medicine Research Division, Korea Institute of Oriental Medicine, Daejeon, South Korea; ^5^The IMOM Korean Medicine Clinic, Jeju, South Korea; ^6^You and Green Korean Medicine Clinic, Daejeon, South Korea

**Keywords:** acupuncture, alternative treatment, case reports, case series, nocturnal crying, night crying, acupuncture for nocturnal crying

## Abstract

**Background:** Nocturnal crying is a common condition in which children intermittently or continuously cry and fuss during the night, at certain times or throughout the night. It is a common pediatric sleep disturbance for which medical assistance is highly sought by parents, and one of the non-pharmacologic treatments for nocturnal crying is pediatric acupuncture. This review aimed to review the literature about the effectiveness and safety of pediatric acupuncture for nocturnal crying.

**Methods:** Literature searches were performed on PubMed, the Cochrane Controlled Register of Trials (CENTRAL), Allied and Complementary Medicine Database (AMED), Chinese National Knowledge Infrastructure Database (CNKI), Wanfang Database, and Chinese Science and Technique Journals Database (VIP), OASIS, the Research Information Service System (RISS), and National Digital Science Library (NDSL) from the available date of inception until December 28, 2020. Two review authors independently screened the titles and abstracts of all relevant articles from the search to select eligible articles. All variants of clinical studies on acupuncture treatment for nocturnal crying, including case reports and case studies, were eligible. Data were independently extracted by two review authors using a standard data extraction form. Retrieved data are presented in a tabular form and narratively discussed.

**Results:** We included 12 studies (10 case series and two case reports) with a total sample size of 2,324 children recruited from the hospital outpatient department. All of the included studies were conducted in mainland China and administered acupuncture as the sole intervention. For the primary outcome, the total efficacy rate of acupuncture treatment for nocturnal crying was reported as 100% in 9 studies, 95% in one study, 94% in another study, and 86% in the remaining study. For the secondary outcome, one study reported a 14% recurrence rate, whereas another study reported an 11% recurrence rate after treatment. There were no follow-ups in most of the studies. None of the studies reported possible adverse events. Most children recovered after one treatment. Generally, the acupoints that were most frequently selected were acupoints EM30 and PC9.

**Conclusions:** This comprehensive review suggested that pediatric acupuncture may be an effective treatment for nocturnal crying, which could be worth investigating further.

## Introduction

Nocturnal crying is defined as episodes of inconsolable crying and fussing during the night in an otherwise completely healthy child. Often, crying occurs at the same time every night, intermittently, or even throughout the night for no known reasons (1). Although nocturnal crying is not listed as a sleep disorder in the International Classification of Sleep Disorders (ICSD-3) (2), this behavior remains a type of pediatric sleep disturbance and can be distressing for parents. Nocturnal crying is a common pediatric sleep disturbance for which medical assistance is frequently sought by parents (3). Not only does nocturnal crying disturb sleep patterns and potentially cause significant stress for parents, but disturbed sleep in children may also result in irritability and behavioral problems (4). Medical assistance for this type of sleep disturbance generally involves conservative behavioral techniques and rarely involves medications (5). Other non-pharmacological alternatives that have been considered include herbal teas, herbal remedies, massage therapy, and chiropractic manipulation.

A non-pharmacologic treatment for nocturnal crying is pediatric acupuncture, a medical technique that stimulates specific acupuncture points in children using needles. Pediatric acupuncture has gradually gained acceptance among parents, even though the evidence supporting the use of acupuncture in children is limited to judging the potential benefits over risks (6, 7). Acupuncture in children generally involves light needling using fine needles or plum-blossom needles or brief pricking using a three-edged needle for mild stimulation (8, 9). As there are many limitations to pediatric acupuncture, such as a lack of cooperation and response-ability in infants and toddlers, acupuncture is not widely used in children for treatment purposes (10). However, anecdotal studies have shown that the effectiveness of pediatric acupuncture is promising (11, 12).

There are currently no systematic reviews concerning acupuncture treatment for nocturnal crying. This study aimed to review the literature about the potential effectiveness of pediatric acupuncture for nocturnal crying based on all relevant cases and case series.

## Methods

This systematic review of the literature identifying all studies on the treatment of pediatric acupuncture for nocturnal crying was reported according to Preferred Reporting Items for Systematic Reviews and Meta-Analyses (PRISMA) guidelines (13).

### Literature Search

Literature searches were performed on PubMed, Embase, the Cochrane Controlled Register of Trials (CENTRAL), Allied and Complementary Medicine Database (AMED), Chinese National Knowledge Infrastructure Database (CNKI), Wanfang Database, Chinese Science and Technique Journals Database (VIP), OASIS, the Research Information Service System (RISS), and National Digital Science Library (NDSL).

Two independent review authors (LA and ES) searched each database from the available date of inception until December 28, 2020, using search terms such as nocturnal crying, night cry, colic, acupuncture, needling therapy, pediatric and traditional medicine. The use of indexing terms, such as Medical Subject Headings (MeSH) terms and other equivalent terms, were applied together with truncation and Boolean searching to achieve wider coverage. References from articles and Google Scholar were also searched to identify other relevant studies. All forms of publication in English, Chinese, and Korean language were eligible for inclusion.

### Inclusion and Exclusion Criteria

#### Study Type

All types of clinical studies, including case reports and case studies, on pediatric acupuncture treatment for nocturnal crying were eligible. Although case studies are subject to publication bias, we decided to include them due to the lack of published studies on pediatric acupuncture for nocturnal crying. Postgraduate theses and dissertations were also eligible. Publications in the form of abstracts, letters, editorials, commentary, and conference proceedings were excluded.

#### Patients

Children of all ages, in particular infants and toddlers, with nocturnal crying treated with pediatric acupuncture were eligible. Children with nocturnal crying due to discomfort, hunger, and other commonly known reasons were excluded. Children with colic were excluded unless the study indicated the time of day when colic occurred as night time or throughout the night.

#### Intervention

All types of pediatric acupuncture were included. There were no limitations on the acupuncture type, application technique, and treatment course. Combined interventions with other alternative therapies that could affect the evaluation of the effectiveness of pediatric acupuncture were excluded.

#### Outcomes

The primary outcome was the efficacy rate of pediatric acupuncture. The efficacy rate was defined as complete resolution of nocturnal crying symptoms after treatment. The secondary outcomes were the recurrence rate and adverse events. All clinically relevant outcomes were eligible, and there were no restrictions for secondary outcomes.

### Study Selection

Two review authors (LA and ES) independently screened the titles and abstracts of all relevant articles from the search to select eligible articles. Subsequently, potentially eligible full-text articles were screened, and the articles were selected according to the inclusion and exclusion criteria. Any disagreement on the eligibility of an article for inclusion in this review was resolved by discussion with all other review authors (HWL, JTK, EK, and MSL).

### Data Extraction and Quality Assessment

Data extracted from the literature included the authors' first name, publication year, study design, sample size, patient demographics (sex and age), acupuncture type, treatment details, treatment length, outcome measures, treatment response (efficacy rate, worsening of symptoms, and recurrence after complete resolution), and adverse reactions. The corresponding authors of a particular included study were contacted for unreported data or missing data. Two review authors (LA and ES) also independently used the Joanna Briggs Institute (JBI) critical appraisal tools for the quality assessment of the included studies (https://jbi.global/critical-appraisal-tools). All queries arising from the two review authors' judgments in the data extraction or the quality assessment were resolved through discussion with all other review authors (HWL, JTK, EK, and MSL).

### Data Synthesis

All the collected data, such as participant baseline characteristics, type of acupuncture treatment, frequency of treatment, and outcome measures, were narratively synthesized. Meta-analysis was not performed, as there are no comparators in the included studies. The details of the study characteristics are presented in a tabular form and narratively discussed.

## Results

### Study Selection

**Figure 1:**
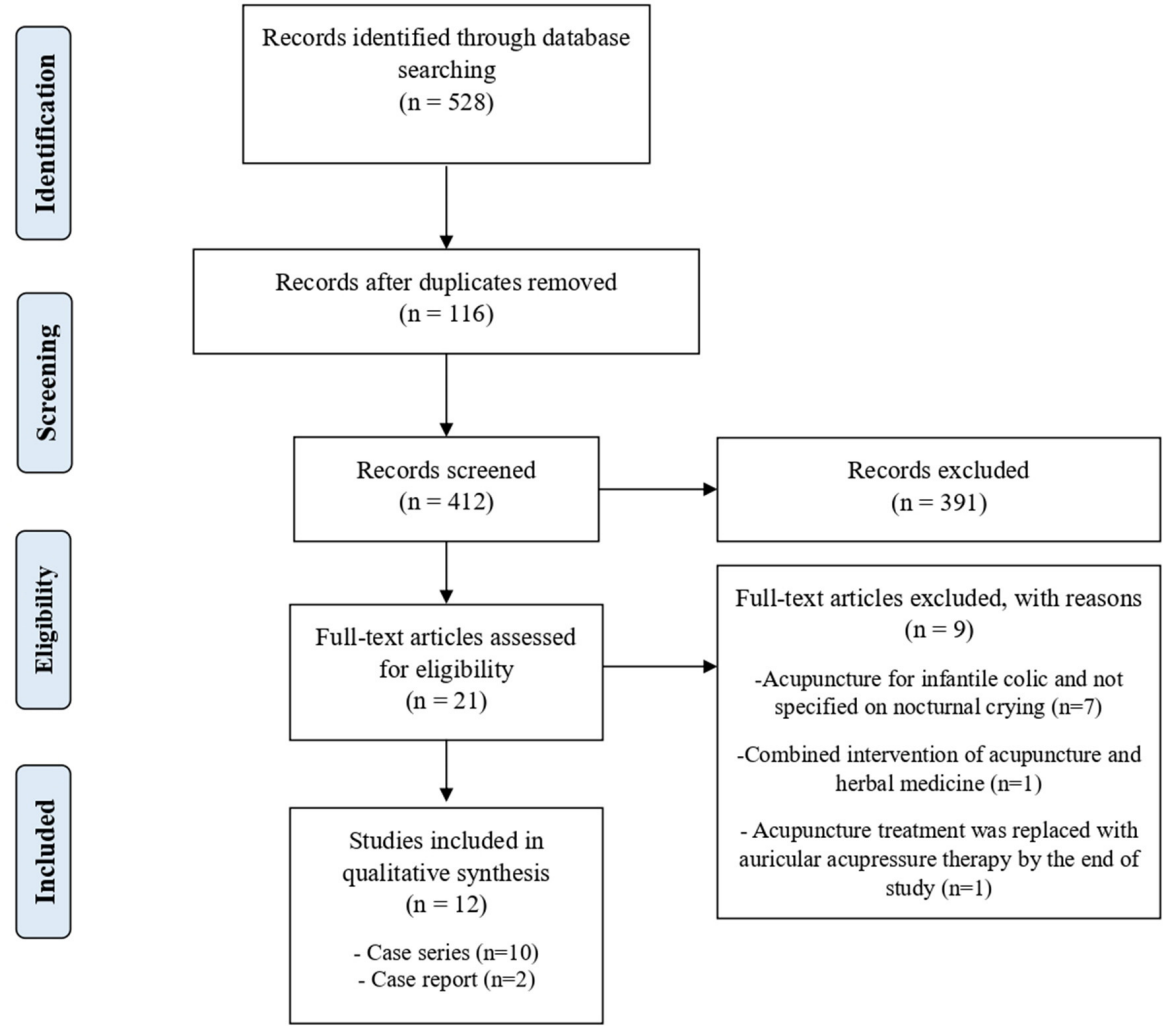
Flow diagram of the study selection process.

Our literature search yielded 528 articles, and 412 articles were screened after removing 116 duplicates. The full texts of 21 eligible articles were assessed. Nine articles were further excluded, as 7 articles did not specifically investigate nocturnal crying but focused on infantile colic in general, 1 article studied the combined intervention with pediatric acupuncture and herbal medicine where herbal medicine may strongly affect the evaluation of the effectiveness of acupuncture treatment, and 1 article replaced the acupuncture intervention with other interventions by the end of their study. In total, we included 12 articles, which included 10 case series (14–23) and 2 case reports (24, 25). All of the included studies were conducted in mainland China. Figure 1 outlines the study selection process.

### Study Characteristics

We identified 2,324 children with nocturnal crying treated with pediatric acupuncture. Of the 2,324 children, 1,088 (47%) were boys, 732 (31%) were girls, and 504 (22%) did not report the sex. The age of the children ranged from 3 days to 7 years old, and the clinical presentation duration ranged from 1 day to 7 months. We could not compute the mean age and disease duration, as a few studies did not provide the relevant information. All children were recruited from the hospital outpatient department, and most of them had histories of treatment for nocturnal crying using standard medication or herbal medicine before receiving pediatric acupuncture. All 12 studies administered pediatric acupuncture as the sole intervention. As most of the included studies provided anecdotal evidence and the treatment course varied across the studies, the results obtained from the included studies could not be further synthesized (meta-analyzed). The details of the study characteristics and treatment regimen are provided in Table 1. Since most of the included studies were case series with aggregated data, individual treatment effects and outcome data were unavailable for each case.

**Table 1:** Summary of study characteristics.

**References**	**Study type**	**Sample size, *N* (boy/girl) age range**	**Treatment regimen (AT type: selected acupoint)**	**Outcome measure**	**Results - treatment frequency: % of children recovered**	**Adverse events (AEs)**	**Follow-up**
Liao and Yu (14)	Case series	118 (51/67) 14 days to 10 mths	**AT once daily for five times treatment** (Plum blossom needle tapping: M-BW-35, HT7, SP6)	1) Efficacy rate	1) 100% - 1 to 2 times: 58%, 3–5 times: 42%	n.r.	n.r.
Liu (15)	Case series	13 (7/6) 2 to 10 mths	**AT once daily for three times treatment** (Three-edged needle pricking: EM30; Filiform needle insertion: PC7)	1) Efficacy rate	1) 100% - 3 times: 100%	n.r.	n.r.
Niu (16)	Case series	12 (n.r.) n.r.	**AT once daily for one-time treatment** (Filiform needle insertion: between PC6 and PC7)	1) Efficacy rate	1) 100%	n.r.	6 mths (no recurrence case)
Lou and Xu (17)	Case series	237 (n.r.) 3 days to 36 mths	**AT once daily for one-time treatment** (Filiform needle insertion: center and both sides of the whole abdomen and back)	1) Efficacy rate2) Recurrence rate	1) 86% 2) 14%	n.r.	n.r.
Zhao (18)	Case series	100 (58/42) 30 days to 36 mths	**AT for one to three times treatment** (Three-edged needle pricking: PC9)	1) Efficacy rate	1) 100% - 1 time: 90%, 2 times: 8%, 3 times: 2%	n.r.	n.r.
Lou (19)	Case series	1,422 (882/540) <2 to 36 mths	**AT once daily for one to three times treatment** (Filiform needle insertion: center and both sides of the whole abdomen and back, BL2, ST36, SP1)	1) Efficacy rate^Δ^2) Recurrence rate^Δ^	1) 100% 2) 11%	n.r.	n.r.
Wang et al. (20)	Case series	34 (19/15) 5–18 mths	**AT for once per 2 days for mild cases and once daily for severe cases for one to four times treatment** (Three-edged needle pricking: EM30)	1) Efficacy rate	1) 94% - 1 time: 24%, 2 times: 35%, 3–4 times: 35%	n.r.	12 mths (no recurrence case)
Wang (21)	Case series	255 (n.r.) 25 days to 7 yrs	**AT once daily for one or two times treatment** (Filiform needle insertion: REN14, EM30, LU11)	1) Efficacy rate	1) 100% - 1 time: 82%, 2 times: 46%	n.r.	n.r.
Cui and Wang (22)	Case series	98 (51/47) 18 days to 11 mths	**AT once daily for six times treatment** [Plum blossom needle tapping: M-BW-35 (especially BL15 and BL19), PC9, ST36, KI1, and extra points (*qi xue*)]	1) Efficacy rate	1) 95% - 1 to 2 times: 26%, 3–8 times: 69%	n.r.	n.r.
Kang (23)	Case series	32 (18/14) 2 days to 10 mths	**AT for one to three times treatment** (Three-edged needle pricking: EM30)	1) Efficacy rate	1) 100% - 1 time: 89%, 2 times: 8%, 3 times: 3%	n.r.	n.r.
Wu (24)	Case reports	2 (2/0) 3–8 mths	**AT once daily for one or two times treatment** (Filiform needle insertion: EM2)	1) Efficacy rate	1) 100% - 1 time: 50%, 2 times: 50%	n.r.	n.r.
Wu (25)	Case report	1 (0/1) 24 mths	**AT twice daily for 1 day** (Filiform needle insertion: PC9, HT7, KI1)	1) Efficacy rate	1) 100%	n.r.	n.r.

*AT, pediatric acupuncture; HM, herbal medicine; n.r., not reported. Δ, reporting errors suspected*.

### Diagnosis and Causes of Nocturnal Crying

There was only one study (23) that reported the diagnostic work-up prior to the diagnosis of nocturnal crying, and the other 11 studies reported cases with clinical manifestations of nocturnal crying. For the diagnostic work-up, Kang's study (23) outlined only the clinical diagnostic criteria, which included intermittently crying and fussing during the night, usually at similar times each night or throughout the whole night, but being fine during the day. Most of the studies did not report the cause of nocturnal crying, except for one study (21) where nocturnal crying in children was due to fright and fear. Additionally, no studies reported if any of the children had neurodevelopmental conditions, where episodes of nocturnal crying may occur as part of their sleep presentation.

### Treatment Characteristics

The pediatric acupuncture administered in all studies varied in the acupoint selection, type of acupuncture needle, treatment technique, and frequency of treatments. Six studies (16, 17, 19, 21, 24, 25) administered pediatric acupuncture by shallowly inserting and manipulating filiform needles in the selected acupoint without leaving the needle inserted. Another four studies (14, 18, 20, 23) required the selected acupoint to be lightly pricked using a three-edged needle, whereas the remaining two studies (14, 22) tapped the selected acupoint using a plum blossom needle. Generally, the acupoints that were most frequently selected were acupoints EM30 and PC9, followed by acupoints ST36, M-BW-35, HT7, and KI1. The treatment frequency ranged from one-time treatment to once-daily treatment for 6 days. All of the studies administered pediatric acupuncture until nocturnal crying symptoms were completely resolved.

### Quality Assessment of Included Studies

The JBI critical appraisal tool for case series and case reports were used to assess the methodological quality of the included study. The JBI case series critical appraisal tool comprised of 10 items related to the risk of bias, adequate reporting, and statistical analysis with a scoring framework of “yes,” “no,” “unclear,” and “not applicable.” On the other hand, the JBI case reports critical appraisal tool included eight items mainly related to adequate reporting, having the same scoring framework as the JBI case series critical appraisal tool. In this review, most of the studies only defined nocturnal crying using clinical manifestation without reporting the diagnostic criteria used. However, the reporting of the patients' clinical information and the result of intervention seemed adequate. The item on statistical analysis was assessed to be “not applicable” as the outcome measure of all studies only included simple percentage calculation and do not require complex statistical analysis. The detailed quality assessments of the included studies are presented in Table 2.

**Table 2:** Quality assessment of included studies.

**Checklist for case series**	**Were there clear criteria for inclusion in the case series?**	**Was the condition measured in a standard, reliable way for all participants included in the case series?**	**Were valid methods used for identification of the condition for all participants included in the case series?**	**Did the case series have consecutive inclusion of participants?**	**Did the case series have complete inclusion of participants?**	**Was there clear reporting of the demographics of the participants in the study?**	**Was there clear reporting of clinical information of the participants?**	**Were the outcomes or follow up results of cases clearly reported?**	**Was there clear reporting of the presenting site(s)/clinic(s) demographic information?**
Liao and Yu (14)	✓	Δ	Δ	Δ	Δ	✓	✓	✓	✓
Liu (15)	✓	Δ	Δ	✓	✓	✓	✓	✓	✓
Niu (16)	✓	Δ	Δ	Δ	Δ	Δ	Δ	✓	✓
Lou and Xu (17)	✓	Δ	Δ	Δ	Δ	✓	✓	✓	✓
Zhao (18)	✓	✓	Δ	Δ	Δ	✓	✓	✓	✓
Lou (19)	✓	✓	Δ	Δ	Δ	✓	✓	✓	✓
Wang et al. (20)	✓	✓	Δ	Δ	Δ	✓	✓	✓	✓
Wang (21)	✓	✓	Δ	Δ	Δ	✓	✓	✓	✓
Cui and Wang (22)	✓	✓	Δ	✓	✓	✓	✓	✓	✓
Kang (23)	✓	✓	✓	✓	✓	✓	✓	✓	✓
**Checklist for case reports**	**Were patient's demographic characteristics clearly described?**	**Was the patient's history clearly described and presented as a timeline?**	**Was the current clinical condition of the patient on presentation clearly described?**	**Were diagnostic tests or assessment methods and the results clearly described?**	**Was the intervention(s) or treatment procedure(s) clearly described?**	**Was the post-intervention clinical condition clearly described?**	**Were adverse events (harms) or unanticipated events identified and described?**	**Does the case report provide takeaway lessons?**	
Wu (24)	✓	Δ	✓	Δ	✓	Δ	Δ	✓	
Wu (25)	✓	✓	✓	✓	✓	✓	Δ	✓	

*✓, “yes”; Δ, “unclear.” Item on “Was statistical analysis appropriate?” was excluded from the table as it is not applicable for all the studies*.

### Measurements of Treatment Effects and Harm

#### Efficacy Rate

The total efficacy rate for pediatric acupuncture treatment for nocturnal crying was reported as 100% in 9 studies (14–16, 18, 19, 21, 23–25), 95% in one study (22), 94% in another study (20), and 86% in the remaining study (17). Of the 2,324 children, nocturnal crying completely resolved in 585 children after one treatment, in 71 children after two treatments, in 22 children after three treatments, in 93 children after <3 treatments, and in 124 children after three or more treatments (Table 3). Data from Lou's study (1,422 children) were not categorized due to suspected reporting errors.

**Table 3:** Summary of treatment frequency and number of recovery.

**Frequency of treatment**	**No. of children recovered**	**Reference**
1 time	585	(16–18, 20, 21, 23)
2 times	69	(18, 20, 21, 23, 25)
3 times	22	(15, 18, 20, 23)
<3 times	95	(14, 22, 24)
≥3 times	124	(14, 20, 22)

*Data from Lou's study (19) with suspected reporting errors was excluded from the above computation*.

#### Recurrence Rate

Only two studies included the recurrence rate as a study outcome. One study (17) reported a 14% recurrence rate, whereas the other study (19) reported an 11% recurrence rate after treatment. Most of the studies did not describe follow-up studies for the recurrence of nocturnal crying, except for two studies. Both studies (16, 20) reported no recurrence cases of nocturnal crying after acupuncture treatment.

#### Adverse Events

None of the studies reported on possible adverse events.

## Discussion

Nocturnal crying is a common clinical condition that is distressing and poorly understood. The common definition of nocturnal crying is children, especially infants and toddlers, intermittently or continuously crying and fussing during the night, at certain times or throughout the night who are stable during the day (1). Nocturnal crying is a specific term used only in traditional medicine and is often referred to as colic in modern medicine. Colic was defined as “crying or fussing more than 3 h of the day for more than 3 days of the week” in Wessel's criteria from 1,954 (26) and “recurrent and prolonged periods of infant crying, fussing or irritability that occur without obvious cause and cannot be prevented or resolved” in the new Rome IV from 2006 (27). The concepts of nocturnal crying and colic are highly similar, where inconsolable crying frequently occurs without known reasons and potential organic causes (28), but nocturnal crying occurs specifically during only the night. However, colic in modern medicine is mostly considered a behavioral condition and can be self-resolving without requiring treatment (29), while nocturnal crying in traditional medicine is pathologized and classified as a disorder requiring treatment (1).

The pathogenesis of both colic and nocturnal crying is yet to be defined. Several studies have shown that colic is associated with gastrointestinal discomforts such as abdominal pain, flatulence, and increased peristalsis (30). However, related studies have not been performed for nocturnal crying due to its traditional medicine characteristics. Nocturnal crying is defined using clinical manifestation and by far only explained using the traditional medicine theory. In this review, children with nocturnal crying of potential gastrointestinal distress are not included as they are part of the exclusion criteria of the included studies. Hence, studies on nocturnal crying evaluated in this review are based on the abovementioned definition of nighttime crying and fussing in children.

The findings of this systematic review suggest that pediatric acupuncture may be an effective treatment for nocturnal crying in children, as the outcomes observed in the literature indicate that acupuncture treatment has a high efficacy rate and potential low recurrence rate. Most children recovered and showed great improvement after receiving one treatment. However, there are no available reports about adverse events and long-term follow-up, which restricts the safety evaluation of pediatric acupuncture for treating nocturnal crying.

This review also provides an overview of the treatment course of pediatric acupuncture for treating nocturnal crying. Few acupoints were selected, and the acupuncture techniques used were shallow needle insertion, brief pricking, or tapping, which result in milder stimulation and are less invasive than acupuncture in adults. This could be an advantage for children who are more sensitive and reluctant to receive treatment. However, the relationship between treatment frequency and treatment effectiveness is unclear, as the available data are insufficient for such an assessment.

The results of this review need to be considered within the context of the study design and diagnosis criteria. Most data that we retrieved were derived from case series and case reports that are subject to publication bias (31). However, the large sample size should be considered, as the current recommendation on the treatment for nocturnal crying is based on a pooled sample size of more than 2,000 children. Nocturnal crying is also a challenging diagnosis to make, with the possibility of variability in the diagnosis criteria (4). Nevertheless, the majority of the included studies made the diagnosis based on the common clinical characteristics of nocturnal crying. Given the rarity of controlled trials on acupuncture treatment for nocturnal crying, we believe that this review provides an important overview of the available literature on this topic.

More randomized controlled trials evaluating the effectiveness of acupuncture for treating nocturnal crying are needed in the future to validate these findings. Reporting guidelines such as “STandards for Reporting Interventions in Clinical Trials of Acupuncture (STRICTA)” (32) should also be implemented while reporting to improve the completeness and transparency of the clinical study. Future considerations should also include developing a diagnostic guideline of nocturnal crying as well as evaluating the cost-effectiveness and ethical concerns of pediatric acupuncture.

## Conclusion

In conclusion, our systematic review provides a comprehensive review of the effectiveness of pediatric acupuncture for treating nocturnal crying. These results suggested that the potential role of pediatric acupuncture for treating nocturnal crying could be worth investigating further.

## Data Availability Statement

The raw data supporting the conclusions of this article will be made available by the authors, without undue reservation.

## Author Contributions

MSL and JTK designed the research. LA and ES performed the research and drafted the manuscript. MSL and EK analyzed the data. HWL reviewed the manuscript. MSL approved and finalized the manuscript. All authors contributed to the article and approved the submitted version.

## Conflict of Interest

The authors declare that the research was conducted in the absence of any commercial or financial relationships that could be construed as a potential conflict of interest.
